# A Review on Ion-Exchange Membranes Fouling during Electrodialysis Process in Food Industry, Part 2: Influence on Transport Properties and Electrochemical Characteristics, Cleaning and Its Consequences

**DOI:** 10.3390/membranes11110811

**Published:** 2021-10-25

**Authors:** Natalia Pismenskaya, Myriam Bdiri, Veronika Sarapulova, Anton Kozmai, Julie Fouilloux, Lassaad Baklouti, Christian Larchet, Estelle Renard, Lasâad Dammak

**Affiliations:** 1 Department of Physical Chemistry, Kuban State University, 149 Stavropolskaya Str., 350040 Krasnodar, Russia; n_pismen@mail.ru (N.P.); vsarapulova@gmail.com (V.S.); kozmay@yandex.ru (A.K.); 2 Institut de Chimie et des Matériaux Paris-Est (ICMPE), Université Paris-Est Créteil, CNRS, ICMPE, UMR 7182, 2 Rue Henri Dunant, 94320 Thiais, France; myriem.bdiri@u-pec.fr (M.B.); julie.fouilloux@u-pec.fr (J.F.); larchet@u-pec.fr (C.L.); e.renard@u-pec.fr (E.R.); 3 Department of Chemistry, College of Sciences and Arts at Al Rass, Qassim University, Ar Rass 51921, Saudi Arabia; bakloutilassaad@yahoo.fr

**Keywords:** ion-exchange membrane, food industry, fouling, transport, mechanical and electrochemical properties, modelling and experiment, cleaning

## Abstract

Ion-exchange membranes (IEMs) are increasingly used in dialysis and electrodialysis processes for the extraction, fractionation and concentration of valuable components, as well as reagent-free control of liquid media pH in the food industry. Fouling of IEMs is specific compared to that observed in the case of reverse or direct osmosis, ultrafiltration, microfiltration, and other membrane processes. This specificity is determined by the high concentration of fixed groups in IEMs, as well as by the phenomena inherent only in electromembrane processes, i.e., induced by an electric field. This review analyzes modern scientific publications on the effect of foulants (mainly typical for the dairy, wine and fruit juice industries) on the structural, transport, mass transfer, and electrochemical characteristics of cation-exchange and anion-exchange membranes. The relationship between the nature of the foulant and the structure, physicochemical, transport properties and behavior of ion-exchange membranes in an electric field is analyzed using experimental data (ion exchange capacity, water content, conductivity, diffusion permeability, limiting current density, water splitting, electroconvection, etc.) and modern mathematical models. The implications of traditional chemical cleaning are taken into account in this analysis and modern non-destructive membrane cleaning methods are discussed. Finally, challenges for the near future were identified.

## 1. Introduction

Ion exchange membranes (IEMs), which are the heart of dialysis and electrodialysis units, are increasingly used in the food industry for the extraction, fractionation and concentration of valuable components, as well as reagent-free control of liquid media pH (whey, wine, fruit juices, etc.) [1,2,3,4,5,6]. Electrodialysis (ED) compares favorably with reverse (RO), direct (FO) osmosis, ultra- (UF) and micro (MF)-filtration by the ability to separate substances not only by particle size, but also by their electrical charge. Many components of whey or blood serum of animals, as well as wines, juices and waste products from these industries change their electrical charge depending on the environment pH. These are proteins, amino acids, anions of polybasic inorganic and organic acids, dyes (anthocyanins) and other polyphenols. Electrodialysis, as well as electrophoresis with IEMs, are some of the processes where this charge (hence, and the efficiency of valuable component separation) can be controlled by reagent less pH change at the surfaces of cation-exchange (CEMs) and anion-exchange (AEMs) membranes [7,8], or by use of bipolar membranes [9]. Such control is carried out by enhancing or suppressing water splitting, the intensity of which depends on the electric field strength and the catalytic activity of membrane fixed groups in relation to the water dissociation reaction [10,11].

Fouling of IEMs is in many respects similar to that described in reviews for RO, FO, UF, and MF processes [12,13]. However, there are also some peculiarities. One of these is steric hindrance during the large organic particles transport in IEMs [14]. The pore sizes of IEM materials, as a rule, do not exceed 40 nm [15,16]. This problem is being actively solved through the use of porous ion-exchange membranes, the introduction of which has been actively carried out recently [17]. Another problem is intense electrostatic interactions between the polar groups of the foulants and the fixed groups of IEMs. Moreover, the degree of this interaction cannot always be predicted based on the pH of the feeding solutions. The reason is acidification (CEMs) or alkalization (AEMs) of the inner membrane solution compared to bathing solution due to Donnan exclusion of hydroxyl ions (CEMs) or protons (AEMs) as co-ions from membranes [18]. These ions are products of the protonation–deprotonation reactions of the above substances when they enter the membrane. In the case of whey and blood serum of animals, which contain only a small amount of aromatic substances, fouling is mainly determined by these electrostatic interactions and the formation of hydrogen bonds between IEMs and foulants [19,20,21]. In the case of wine, juices, and tea, π-π (stacking) interactions between the aromatic rings of polyphenols (PP, contained in these liquid media) and the aromatic matrix of IEMs are added to the above mentioned interactions [22,23,24,25]. Moreover, even a small amount of PP can trigger the formation of colloidal aggregates in the pores and on the membrane surface.

This review focuses on the analysis of modern scientific publications, which investigated the effect of foulants and chemical cleaning agents on the structural, transport, mass transfer and electrochemical characteristics of cation and anion exchange membranes used in dialysis and electrodialysis processing of liquid media in the food industry. Much attention is paid to modern theoretical concepts and mathematical models that can be used to interpret experimental data and predict the behavior of fouled IEMs. The implications of traditional chemical cleaning are taken into account in this analysis and modern non-destructive membrane cleaning methods are discussed. 

## 2. Impact of Traditional Cleaning Methods on the Chemical Structure of IEMs

**Figure 1:**
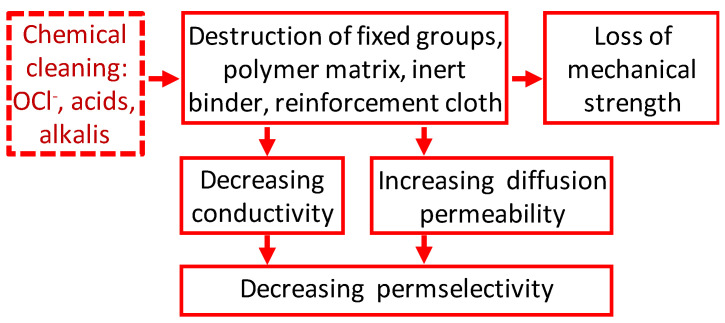
Influence of traditional chemical cleaning on the structure, mechanical strength and transport characteristics of ion-exchange membranes.

As mentioned above, the vast majority of liquid media in the food industry are a breeding ground for microorganisms. This fact, together with the need to counteract the growth of colloidal structures inside IEM and on its surface, necessitate regular cleaning of membrane stacks of ED apparatuses [26]. Most often, HCl, NaOH, hypochlorides and other oxidants (peracetic acid, and P3 Active Oxonia^®^ (Ecolab, Saint Paul, MN, USA) solutions) are used for these purposes [27,28,29,30,31,32,33,34]. Moreover, cleaning is carried out at a high temperature (60–90 °C) [35,36,37]. A number of articles have been devoted to the influence of these procedures on the structure and chemical properties of membranes. The results of these works can be briefly summarized as follows (Figure 1). Thermochemical desulfonation (detachment of sulfonate groups) in aqueous solutions is possible in the case of CEMs [38]. Fixed amino groups of AEMs are much more susceptible to destruction under cleaning conditions. Methylated ammonium-type fixed groups are the least stable in an alkaline environment [39] or in the presence of hypochlorite and hydrogen peroxide [27]. As a result of the reaction called Stevens rearrangement [40], as well as other reactions described in detail in [41], some of the quaternary amino groups are converted into secondary and tertiary amines. In addition, there is the detachment of fixed groups, rupture of the carbon chains of the polymer matrix, and partial destruction of the copolymer of polystyrene and divinylbenzene, which make up the ion-exchange matrix of most aromatic membranes [42]. The result of such reactions is the appearance on the surface and inside the IEMs of cavities, the walls of which do not have an electric charge. Hydroxyl ions influence not only on the ion-exchange material, but also on polyvinyl chloride, PVC (an inert filler and reinforcing cloth of most membranes made by the paste method [43]), according to the 2E elimination reaction [44] mechanism. Hydroxyl ions pull out β-hydrogen from PVC; the chloride ions are eliminated and double bonds are formed, forming black polyenes [45,46,47]. Such degradation is more significant for AEMs [48], because hydroxyl ions are counter ions, and their concentration in the internal solution of AEMs is high. In CEM, for which hydroxyl ions are co-ions and are excluded from the membrane due to the Donnan effect, PVC degradation is observed only at very high external solution pH [46,48]. In addition, even at neutral pH of the solution adjacent to the membrane, under the action of a high electric field strength, free radicals are formed on the PVC surface, which initiate the breaking of macrochains with the formation of oxidation products [49]. Grains of PVC, not fixed by the ion-exchange material (which is the cause of the IEM destruction), are washed out by the solution that flows over the membrane. Operation of AEM in intense current regimes of ED tartrate- and phosphate-containing solutions processing intensifies the process of AEM degradation in comparison with NaCl solutions [50].

Vasil’eva et al. [38] found that the contact of heterogeneous membranes with acid, alkali or even water at high temperatures leads to an increase in the gaps (macropores) between the beads of the ion exchange resin and low-pressure polyethylene, which is an inert binder in heterogeneous CEMs and AEMs. The polyethylene degradation in water occurs due to the washing out of the antioxidants [51] and hydrolytic oxidation of the polymer with the formation of carboxyl groups [52]. Dissolution of reinforcing cloth made of nylon and similar materials is possible by treating membranes with acids [38].

Thus, the cleaning by traditional chemical reagents leads not only to the partial loss of the AEM and CEM fixed groups, but also to the formation of cavities and/or microcavities on the surface and in the volume of the IEM. These cavities are often not electrically charged and can be filled with colloidal particles of foulants. As will be shown in the following chapters, the application of traditional cleaning with oxidizing agents and other harsh chemicals sometimes does as much detriment to IEM performance as fouling. Moreover, the consequences of such hard cleaning contribute to the intensification of fouling. If membranes are used for a long time in industrial electrodialysis, it is not always possible to separate the effect of fouling and cleaning on equilibrium (water content and exchange capacity) and transport (conductivity, diffusion permeability, selectivity) characteristics of IEMs, as well as on their behavior in an electric field. Therefore, we will analyze the impact of both fouling and hard cleaning in the following chapters.

## 3. Water Content and Exchange Capacity of Fouled IEMs

The ion exchange capacity, IEC, is defined as the number of functional sites (in mmole) per mass (in gram) or volume (in cm^3^) of dried or swollen membrane [53]. In all publications related to the study of IEMs fouling, organic matter always has a tendency to decrease the IEC by total or partial inhibition of the functional sites and accumulation of organic particles that form aggregates of more or less dense colloidal particles [21,54,55]. In addition, cleaning of membrane stacks at high temperatures and using oxidizing agents result in the elimination of fixed groups or, in the case of AEM, the conversion of strongly basic quaternary amino groups to weakly basic secondary or tertiary amino groups.

As discussed in Section 2, the degree of reduction in IEC of fouled membranes as compared to a pristine one is determined by the cleaning method (chemical nature of the substances used, temperature); the chemical nature of the fixed groups, polymer matrix and IEM inert filler; pH of the working solution; and current modes (under-limiting, overlimiting) of the ED operation.

**Figure 2:**
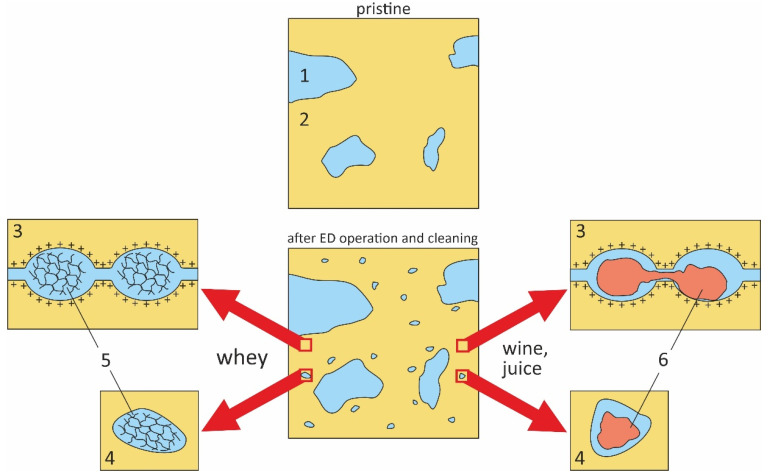
The proposed structure of AEMs that contain PVC as an inert filler, before and after their use in the ED process of wine tartrate stabilization: (1) macro-defects, filled with an external solution; (2) nanoporous medium; (3) nanopore with fixed groups on the walls, filled with an external solution; (4) nanopore with non-electrically charged walls filled with an external solution; (5) reticulated hydraulically permeable colloidal structures; (6) hydraulically impermeable colloidal aggregates. Adapted from [58,59].

In the processes of milk (acid or sweet whey, whey protein isolate or hydrolysate, etc.) or animal serum electrodialysis, the decrease in IEC is mainly determined by electrostatic interactions of membranes with highly hydrated colloidal structures that form proteins, carboxylic acids, amino acids, and mineral salts (sodium, potassium, calcium, phosphorus and magnesium). To a greater extent, the IEC of CEMs decreases in acidic solutions, while the IEC of AEMs increases in alkaline and neutral solutions [26,56,57,58]. These colloidal (network) structures fill both the pores of the ion-exchange material (the walls of which contain polar fixed groups) and the pores with non-charged walls (the reasons for the appearance of these pores were discussed in Section 2), contributing to an increase in water uptake. The structure of such fouled membranes is shown in Figure 2. The changes in IEC, water uptake and membrane thickness are not dramatic. For example, Garcia-Vasquez et al. [58] observed a 20% decrease in IEC, 30% increase in water uptake and <2% increase in thickness of the AMX-Sb anion exchange membrane (Astom, Osaka, Japan) after several thousand hours of the sweet milk whey ED demineralization.

The scenario and consequences of IEM fouling change fundamentally in the case of juices, tea and wine that contain polyphenols (PP). As mentioned in [18,22,23,24], the main role in fouling is played by the π-π (stacking) interactions of polyphenols with each other and with the IEM materials. Colloidal particles formed inside the pores are compacted during long-term use of IEM (also, as a result of cleaning) and turn into hydraulically impermeable agglomerates. As a result, the effective IEM pore diameter decreases [22,23,59,60,61,62,63] (Figure 2). 

For example, Ghalloussi et al. [59] have shown that the decrease in IEC of Neosepta CEMs (Astom, Osaka, Japan) after several thousand hours of operation in ED is from 3% to 84%. In this and in later works [22,23,59,60,61], it was found that the most significant decrease in IEC is observed for membranes with an aromatic ion-exchange matrix and is accompanied by an insignificant (7–16%) decrease in water uptake. On the contrary, membranes with an aliphatic ion-exchange matrix demonstrate a less noticeable decrease in IEC, but their water uptake can increase by 30–40% (taking into account the formation of large cracks and cavities filled with water). The thickness of some AEMs and CEMs increased by a factor of 1.6–2.1 [22,59]; the linear dimensions of other membranes do not undergo significant changes [18,22,60,61,62,63,64]. For example, after 2600 ± 100 h of operation in industrial ED processing of polyphenol (PP)-containing liquids, the thickness of aromatic membranes made by the paste method increases by 52% (CMX-Sb) and 11% (AMX-Sb). The reasons for the different behavior of these membranes are the ability of anthocyanins and other PPs to change their electrical charge depending on the environment pH. Industrial ED processing of wines and juices is carried out at pH < 3.5, where anthocyanins and other polyphenols are typically cations (PP^+^). Low pH values within the cation exchange membrane contribute to maintain the PP^+^ positive electrical charge as it enters CEMs [18,23]. The PP^+^ cations enter the electrostatic interactions with the fixed groups of CEMs, which are negatively charged, and screen these groups. In contrast, the pH of the internal solution of AEMs is neutral or slightly alkaline [18,60]. Upon entry into AEMs, PP^+^ lose their electrical charge or become anions that do not enter the electrostatic interactions with positively charged fixed groups of AEMs or are excluded from them as co-ions. 

## 4. Transport Characteristics of Fouled IEMs

Electrical resistance (*R_m_*) or conductivity (*κ_m_*), as well as diffusion permeability of IEMs, determine the energy consumption for the ED process and the resulting concentration of desalinated and concentrated solutions. Knowing these parameters, it is possible to calculate the counterion and co-ion transport numbers in IEM ($t_{m1}$ and $t_{mA}$, respectively) [65], that determine membrane selectivity [66]:

(1) $$t_{m1}=\frac{1}{2}+\sqrt{\frac{1}{4}-\frac{\left(z_{1}\left|z_{A}\right|\right)P^{*}F^{2}C}{\left(z_{1}+\left|z_{A}\right|\right)RT\kappa_{m}}}\;\;,\;t_{mA}=1-t_{m1}$$

where *z*_1_ and *z_A_* are the charge numbers of the counterion and co-ion, respectively; *C* is an electrolyte concentration; *F* is the Faraday constant; *R* is the gas constant; *T* is the temperature. The relation between the differential (or local) diffusion permeability coefficient, P*, and the integral diffusion permeability coefficient of membranes (*P_m_*) is given as $P_{m}=\frac{1}{C}\int_{0}^{C}P^{*}dC\;$[67,68]. It leads to the following equation [69]:

(2) $$P^{*}=P_{m}+C\frac{dP}{dC}$$

In practice, for calculation of P*, it is more convenient to use the relationship between P* and *P* in the form [69]:

(3) $$P^{*}=P_{m}\left(\beta +1\right)$$

with *β = dlogP_m_/dlogC*, which is the slope of the *logP_m_* vs. *logC* dependence. The integral diffusion permeability coefficient of membranes (is usually defined as [68]:

(4) $$P=j\frac{d}{C}$$

where *j* is the electrolyte flux density. This electrolyte diffuses through IEM from the compartment with electrolyte solution with concentration *C* into the compartment with initially distilled water; *d* is the membrane thickness. Experimental methods for determining the resistance (electrical conductivity), diffusion permeability and selectivity of IEM are summarized in reviews [66,68,70]. For example, the differential method with a clip cell [71,72] seems to be the simplest and most reliable to determine the electrical conductivity after removing the IEM from the ED apparatus. The electrolyte solutions used in most publications are mainly NaCl or KCl solutions at 0.1–1.0 M [67,68,73,74,75], or even at lower concentration [70,72] for both homogeneous or heterogeneous IEMs. Membranes, after their operation in the food industry, are also tested in these solutions [23,60,61,62,64,76]. This opens the possibility of using known mathematical models to determine the structural parameters of IEMs or the effect of fouling on the development of water splitting and electroconvection. This is due to the fact that most of the already developed models do not take into account the possible interactions of foulants with the matrix and fixed groups of membranes, as well as the possible change in the electric charge of amino acids, polybasic acid anions, anthocyanins, etc. in IEMs as compared to the external solution [18,23,77,78].

Monitoring the resistance of the membrane stack or individual membranes in situ (in operando) is carried out by applying Ohm’s law when a constant electric field is applied [79] or by periodically measuring the impedance of the membrane system in an alternating current mode in the high frequency range (>1 kHz) [80,81]. To determine the resistance of the membrane system, the initial sections in current–voltage characteristics and ohmic sections in chronopotentiograms [82,83] are used. Another option is to find the length of the segment on the abscissa axis, cut off by the high-frequency arc of electrochemical impedance spectrum represented in the complex plane [79,80].

Voltage and current readings from the indicator on the power supply [79] are commonly used in ED industrial application. However, the values obtained in this way include not only the actual IEM resistance, but also the resistance of the diffusion layers adjacent to the membrane, which is controlled by the degree of concentration polarization development (see Section 5). Therefore, such assessments should be treated with caution.

### 4.1. Modelling 

**Figure 3:**
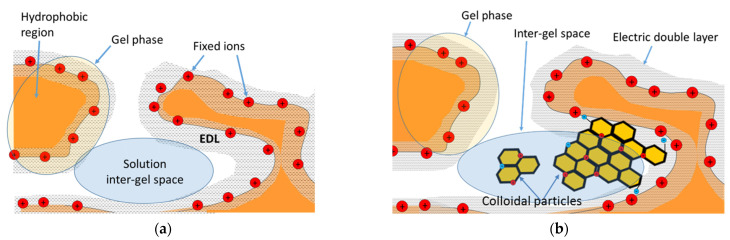
Schematic IEM structure represented in terms of microheterogeneous model [68] (**a**), and the same model, which takes into account colloidal particles (**b**) formed by polyphenols. EDL is the electric double layer. Adapted from [60].

Most of the “classical” [84,85,86,87] and modern [88,89,90,91,92] models are intended to describe transport phenomena in ideally homogeneous IEMs. The microheterogeneous model developed by Gnusin, Nikonenko, Zabolotskii et al. [68,74,93] seems to be the most suitable for real membrane systems. It is widely used in the literature [14,48,70,75,94,95,96,97,98,99] to interpret the experimental concentration dependences of membrane conductivity, diffusion permeability, and to determine the relationship “structure–transport properties” of IEMs. The model is based on a simplified representation of the IEM structure, according to which the membrane can be considered as a multiphase (in the simplest case, two-phase) system. The properties of such a system are determined by the properties of individual phases, which is consistent with the fundamentals of the effective medium theory [100]. The gel phase (denoted by the index g) is a microporous swollen medium. It includes a polymer matrix carrying fixed groups, as well as a charged solution of mobile counterions (and, to a lesser extent, co-ions), which balance the charge of fixed groups. There are two regions within the gel phase. A “pure” gel consists of an electrically charged double electrical layer and side polymer chains with hydrated fixed groups. The hydrophobic regions are formed by agglomerates of polymer chains that do not contain fixed groups, as well as an inert binder and reinforcing cloth. The second phase, the inter-gel space (denoted by index “int”) is an electrically neutral solution (identical to the external solution). It is located in the central part of the meso- and macropores, and also fills in the membrane structural defects. A membrane fragment that includes both phases is shown in Figure 3.

In accordance with the approach that combines the effective medium theory and nonequilibrium thermodynamics [101], the flux of ions of sort *i* (*j_i_*) in a two-phase IEM is proportional to the gradient of the electrochemical potential $d\mu_{i}/dx$ [68,102]:

(5) $$J_{i}=-L_{i}^{*}d\mu_{i}/dx$$

where $L_{i}^{*}$ is the effective conductivity coefficient characterizing a multiphase medium, which is equal to:

(6) $$L_{i}^{*}={\left[f_{1}{\left(L_{i}^{g}\right)}^{\alpha }+f_{2}{\left(L_{i}^{int}\right)}^{\alpha }\right]}^{1/\alpha }$$

The conductivity coefficients $L_{i}^{g}$ and $L_{i}^{int}$ refer to the gel phase and the inter-gel solution phase, respectively. The structural parameter *α* takes values from 1 to −1, respectively, with a parallel and sequential arrangement of phases; the sum of the volume fraction of the gel phase (*f*_1_) and the inter-gel space (*f*_2_) is equal to one (*f*_1_ + *f*_2_ = 1). The values $L_{i}^{g}$ and $L_{i}^{int}$ are determined by the ion diffusion permeability coefficients $D_{i}^{g}$ and $D_{i}^{int}$, and ion concentration, *C*, in the corresponding phase:

(7) $$L_{i}^{g}=D_{i}^{g}C_{i}^{g}/RT$$

(8) $$L_{i}^{int}=D_{i}^{s}C_{i}^{s}/RT$$

The exchange capacity of the membrane gel phase (the concentration of fixed groups in the gel phase), *Q_g_*, is related to the membrane exchange capacity, *Q*, by the equation: *Q_g_* = *Q*/*f_1_*. From Equations (5)–(8), simple expressions are obtained to determine the membrane conductivity (*κ_m_*), diffusion permeability (*P_m_*) and counterion transport numbers (*t_m−_*) (for example, in AEM):

(9) $$\kappa_{m}=\left(z_{+}L_{m+}+z_{-}L_{m-}\right)F^{2}$$

(10) $$t_{m-}=\frac{L_{m-}}{L_{m-}+L_{m+}}$$

(11) $$P_{m}=2t_{m-}L_{m+}\frac{RT}{C}$$

where index “+” relates to a cation (co-ion in the AEM).

The simple relationship between the membrane conductivity (*κ_m_*) and the conductivities of the gel phase (*κ_g_*) and electroneutral solution (*κ_s_*) filling the inter-gel spaces is of the greatest interest:

(12) $$\kappa_{m}={\left(f_{1}\kappa_{g}^{\alpha }+f_{2}\kappa_{int}^{\alpha }\right)}^{1/\alpha }$$

If |*α*| is not too far from zero (<0.2), and the external solution concentration is in the range 0.1 *C*_iso_ < *C* < 10*C*_iso_ (*C*_iso_ is the electrolyte concentration at the isoconductance point: *κ_m_* = *κ_g_* = *κ_s_*), Equation (12) may be approximated as:

(13) $$log\left(\kappa_{m}\right)\approx f_{2}\log\left(C\right)+const$$

where const ≈ (1 − *f*_2_)log(*κ_g_*).

It is believed that in all the cases under consideration, fouling leads to a decrease in the membrane IEC. In the case of formation of hydraulically permeable reticular colloidal structures (milk whey) [76] in the pores:

(14) $$L_{i}^{int}=\left(\gamma D_{i}^{s}\right)C_{i}^{s}/RT$$

where *γ* is a coefficient reflecting the ratio of ion mobility in the intergel space of fouled membrane and in an external solution.

In the case of contact of IEMs with polyphenol-containing solutions (wine, juices, tea extracts), it is believed that dense hydraulically impermeable conglomerates of colloidal particles (denoted by index “*cp*”) are formed in the meso- and macropores, but do not penetrate into the nanopores. These changes in the structure can lead to a possible change in the ion transport path, which is manifested in a change in the structural parameter from α to β. In this case, from Equation (12), it is easy to obtain an equation for *κ_m_* [60]:

(15) $$\kappa_{m}={\left[f_{1}\kappa_{g}^{\alpha }+f_{2}{\left(f_{2s}^{int}\right)}^{\alpha /\beta }\kappa_{s}^{\alpha }\right]}^{1/\alpha }$$

Equation (15) can be also approximated in the same way as Equation (12):

(16) $$log\left(\kappa_{m}\right)\approx f_{2}{\left(f_{2s}^{int}\right)}^{\alpha /\beta }log\left(C\right)+const$$

A similar approach was used to describe the transport characteristics of IEMs with conglomerates of various nanoparticles immobilized in their meso- and macropores [103].

The use of the basic [68] and modified microheterogeneous model [60,76,103] develops a theoretical basis for explaining the effect of foulants on the membrane transport characteristics and makes it possible to predict the tendency of IEM to fouling depending on membrane structure and component composition of processed solutions in the food industry.

### 4.2. Interpretation of Experimental Data Using a Microheterogeneous Model

IEMs conductivity, as a rule, decreases after prolonged exposure to liquid media typical for the food industry [22,23,59,60,61,104,105]. In the case where a membrane is in contact with amino acids, carboxylic acids or anions of polybasic inorganic acids, the decrease in conductivity is not dramatic [58,104] and can be reversible if a dense layer of proteins or mineral precipitate does not form on the IEM surface [28,29,106]. The decrease in *κ_m_* is most often caused by electrostatic interactions and hydrogen bonds of foulants with fixed EIM groups.

The *f*_2_ values of IEMs increase as a result of the polymer matrix stretching when strongly hydrated ions enter it [16], or due to its destruction as a result of cleaning (see Section 2), as well as due to the operation of membranes in intensive current regimes. For example, Garcia-Vasquez et al. showed that after several thousand hours of the Neosepta AEM (Astom, Osaka, Japan) used in ED demineralization of whey, *κ_m_* decreases by a factor of 1.3 and *f*_2_ increases by 25% as compared to pristine AEM.

In the case of polyphenol containing liquid media, the membrane conductivity (and other transport characteristics) undergo more significant changes. For example, after exposure to green tea, *κ_m_* of AEMs dropped by at least 50% (AMX-Sb, AFN, Astom, Osaka, Japan, and PC-400 D) [107,108]. After contact with wine and cranberry juice, *κ_m_* of AEMs decreased by a factor of 1.6–1.9 [59], 4 [22] (AEMs Neosepta), 3 [105] (MA-41P, Shchekinoazot, Pervomaisky, Russia) and 4 (AMX, AMX-Sb, Astom, Osaka, Japan) times. The conductivity of CEMs decreased by 2 [22,61], 1.1 (MK-40, Shchekinoazot, Pervomaisky, Russia), 1.2 (Fuji CEM Type II, Fujifilm, the Netherland; CSE-fg, Astom, Osaka, Japan) and 1.7 (CJMC-5, ChemJoi, Hefei, China) [23] times. The decrease in conductivity is primarily caused by the loss of IEM in exchange capacity due to not only electrostatic, but also π-π (stacking) interactions of foulants with ion-exchange materials.

It is important to note that the experimental data treatment on conductivity using a microheterogeneous model demonstrates a clear decrease in the volume fraction of the gel phase *f*_2_ in the case of CEMs. This result provided additional evidence for the formation of agglomerates of hydraulically impermeable colloidal particles in the pores of cation exchange membranes.

**Figure 4:**
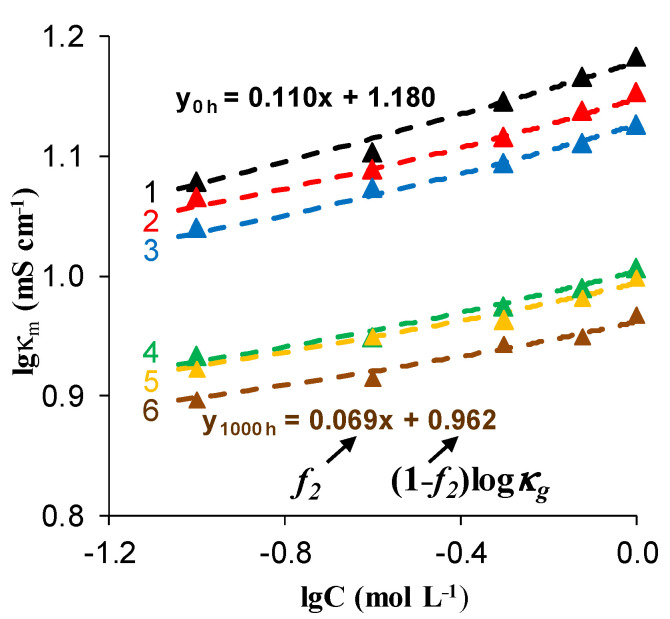
The conductivity of the AEMs soaked in the synthetic polyphenol-containing solution vs. NaCl concentration. Markers show the experimental data measured for sample soaking duration 0 (1), 24 (2), 100 (3), 500 (4), 750 (5) and 1000 (6) hours; dashed lines are the results of calculations according to the modified microheterogeneous model. Adapted from [60].

As for AEMs, in some studies [59,105], an increase in *f*_2_ is observed, while in [22] the volume fraction of the intergel space decreases. To clarify the reasons for this seeming inconsistency, Bdiri et al. studied the fouling kinetics of aromatic AEM soaked in synthetic solution which contained tartaric, acetic, lactic acids, KCl, CaCl_2_ and a high concentration of polyphenols (5 g.L^−1^), sufficiently greater than that in juices and wine [109,110]. Processing these data (Figure 4) using a modified microheterogeneous model showed that both the conductivity and the volume fraction of the gel phase decrease with an increase in the time of membrane contact with the phenol-containing solution (Table 1). 

Pristine membrane, which have not been soaked in the model solution (h = 0) does not contain colloidal particles. However, *f*_2s_ = *f*_2_ = 0.09 values are slightly lower than the apparent volume fraction of the inter-gel spaces, *f*_2app_ = 0.11 (the slope of the log(*κ_m_*) vs. log(*C*) dependence, Figure 4). Colloidal particles are formed in the inter-gel spaces with an increase in soaking time. They displace a part of the electroneutral solution that results in decreasing *f*_2*s*_, and, as a consequence, *f*_2app_. A part of AEM functional groups becomes blocked by the colloidal particles that lead to IEC decreases with soaking duration. The increase in counterion diffusion coefficient in gel phase $D_{-}^{g}$ (Table 1) should be due to an increase in membrane swelling degree. This parameter can be estimated by the ratio of the fouled and pristine AEMs thickness, *d/d_h_ =* 0. Higher swelling indicates an increase in the size of micropores that promotes counterion mobility. Apparently, higher swelling of IEMs in the considered liquid media is caused by relatively small and highly hydrated [66] organic acid species, such as tartaric acid. 

**Figure 5:**
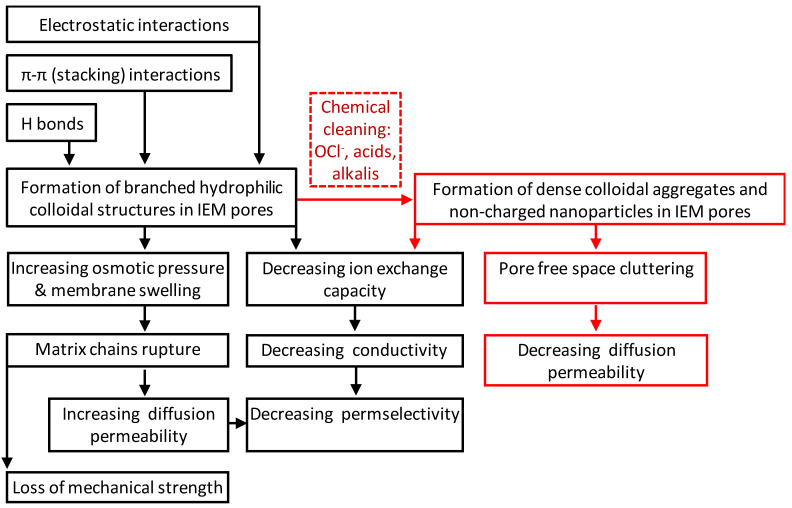
The main types of interaction of polyphenols with ion-exchange membranes and the effect of this fouling on the transport characteristics of IEMs.

Faster destruction of anion-exchange membranes during cleaning in ED industrial processes and in the case of application of intensive current modes is due to the more alkaline media inside AEM compared to CEM (see Section 2). If an increase in *f*_2_ caused by this factor is not compensated by a decrease in the inter-gel space due to the formation of agglomerates of colloidal particles, we observe an increase in the volume fraction of the gel phase and an increase in the AEM diffusion permeability. Note also, the diffusion permeability of AEMs in juices and wine may increase slightly if colloidal particles do not form agglomerates (no cleaning) and are completely or partially destroyed by concentrated saline solutions, which are usually used to study diffusion permeability [76,103]. The main types of interaction of polyphenols with ion-exchange membranes and the effect of this fouling on the transport characteristics of IEMs are summarized in the Figure 5.

## 5. Effect of Fouling on IEMs Behavior in an Electric Field

### 5.1. Theoretical Background

Many of the researchers note that fouling leads to a decrease in the current efficiency (*η*) in ED of liquid media (including in the food industry) [108,111,112,113,114,115]. It is known [116,117] that the current efficiency of the target component (for definiteness, the salt anion) is determined by the difference between the effective transport numbers of this anion through AEM (as a counterion) and through CEM (as a co-ion), which form the ED desalination channel. For simplicity, we neglect the transfer of the target component through CEM and consider a solution that contains only one salt, for example, NaCl. In this case, the current efficiency is close to the value of the counterion effective transport number through AEM (*T*_1_), which is equal to the ratio of the counterion partial current density, *i*_1_, to the total current density in the membrane system, *i*:

(17) $$\eta \approx \frac{i_{1}}{i}=T_{1}=1-T_{A}-T_{w}$$

According to Equation (17), the more the effective counterion transport number (*T*_1_) differs from unity, the higher the effective transport numbers of co-ions—salt cations (*T_A_*) and water splitting (*T_w_*) products, for example, hydroxyl ions (*T_OH_*) (generated at the AEM/depleted solution interface). If the difference in the concentration of the target electrolyte at the releasing and receiving membrane surfaces is not too large, then *T_A_ ≈ t_mA_* [118]. According to Equation (1), the *t_mA_* value is determined by the ratio of the conductivity to the differential coefficient of the membrane diffusion permeability. Thus, a decrease in conductivity and an increase in membrane diffusion permeability due to fouling (see Section 4) can actually lead to a decrease in current efficiency.

The effect of fouling on electric current-induced phenomena, i.e., water splitting (WS) and electroconvection (EC), has been intensively studied recently. Reviews of works devoted to these phenomena can be found in [11,119,120,121]. Interest in these phenomena stems from the fact that they affect (a) the value of *T_w_* (*T_OH_* for AEM and *T_H_* for CEM); and (b) precipitation and fouling. WS causes a local pH shift at the IEM surfaces [122], which leads to intensification of precipitation or, on the contrary, dissolution of already formed precipitates [123]. EC intensively mixes the solution at the IEM / depleted diffusion layer interface, counteracting the fixation of precipitates on the membrane surface [124] and shifting WS toward higher potential drops [125].

The term “water splitting” implies the generation of H^+^ and OH^–^ ions at the IEM/depleted solution interface or at the bipolar CEM/AEM interface with the participation of fixed membrane groups. The catalytic activity of fixed groups in relation to water dissociation is determined by the values of the protonation–deprotonation reactions of these groups, the electric field strength at the membrane/solution interface and the rate of reaction product (H^+^ and OH^–^ ions) removal from the reaction zone [10,126,127,128] with a thickness of several nanometers. 

It is known [10,126] that carboxyl, phosphoric acid groups, as well as secondary and primary amines, have high catalytic activity, while fixed sulfonate groups and quaternary ammonium groups have low catalytic activity with respect to WS. A high electric field strength promotes a special orientation of water molecules favorable for the proton transfer and “stretching” of the H–OH [129] bonds.

In the case of monopolar membranes, the electric field strength required for WS is achieved at current densities (*i*) close to the limiting value (*i_lim_*) or higher [119,126,128]. An increase in the current density, as well as the presence of cation-exchange and anion-exchange layers that form a bipolar junction, contribute to an increase in the electric field strength in the reaction zone and accelerate the removal of H^+^ and OH^–^ ions from the reaction zone [119,126,127].

It is important to note that many of the liquid media components in the food industry (ammonium, amino acids, polybasic carboxylic and inorganic acids, etc.) are an additional source of protons or hydroxyl ions at the IEM/solution interface [10,77,78,130,131,132]. The point is that they are involved in protonation–deprotonation reactions in solution and in the membrane. If the products of these reactions (H^+^ or OH^–^ ions) are co-ions, they are excluded from the IEM due to the Donnan effect.

This phenomenon intensifies with an increase in the current density due to a decrease in the concentration of the depleted solution at the IEM surface [78,133]. It is known [10,126,134] that some of the precipitated substances, for example, magnesium hydroxide (which is a traditional component of milk whey) participate in protonation–deprotonation reactions and intensify the generation of H^+^ and OH^–^ ions.

The term “electroconvection” denotes a quite diverse phenomena, which manifest themselves in the appearance of vortex structures in a depleted solution at the surface of monopolar IEMs. An indispensable condition for the EC development is the presence of a tangential component of the electric field, as well as an electric double layer (under-limiting current regimes) and/or an extended space charge region (over-limiting current regimes) [135,136,137].

Therefore, the degree of EC development depends on the surface charge [138], as well as its electrical (alternation of conductive and non-conductive areas) [136,139,140,141] and geometric (waviness, roughness) [135,137,140,142] inhomogeneity. Besides, the development of EC is enhanced by an increase in surface hydrophobicity, which facilitates fluid slip along the IEM/solution interface [124,138].

### 5.2. Voltammetry 

**Figure 6:**
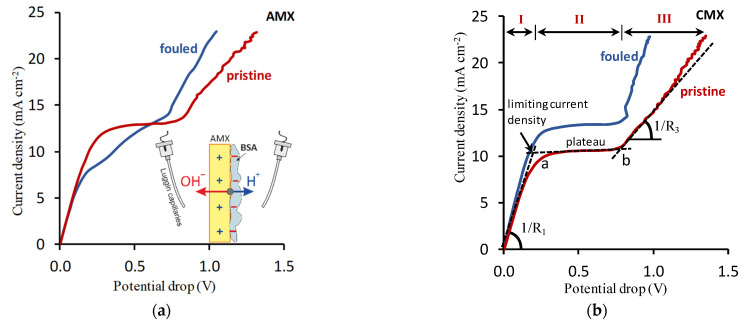
CVC of pristine and fouled by BSA anion exchange AMX (**a**) and cation exchange CMX (**b**) membranes. Insert in (**a**) shows the scheme of the mechanism of water splitting enhancement due to the formation of a bipolar junction between positively charged fixed AMX groups and negatively charged BSA. Explanations can be found in the text. Constructed using data from [146].

Current–voltage characteristic (CVC) analysis is the most common method for assessing the effect of fouling on WS, EC, electrical resistance, *T*_1_ and IEM conductive surface area [64,111,143,144,145,146,147,148,149]. As a rule, these curves (Figure 6) are obtained in flow pass or non-flowing ED laboratory cells, in which the electric current increases slowly (0.01 mA/s) and the potential drop between the Luggin capillaries is recorded. Luggin capillaries are in solution on both sides of the membrane under study. The group of researchers led by Seung-Hyeon Moon was apparently one of the first to use independent experiments to interpret the CVC, and proposed an algorithm for assessing the effect of fouling on IEM behavior in an electric field [111,146,150,151], which is now used by many researchers. For example, in [146], studies of cation and anion exchange membranes CMX and AMX (Astom, Osaka, Japan) were carried out in a solution of a strong electrolyte (0.01 M KCl) and in the same solution containing a foulant (0.01 M KCl and 1% BSA). The authors [146] concluded that change in the slope of the initial section of the CVC (Region I in Figure 6b) obtained in a solution with foulant vs. a solution that does not contain it, show the foulant effect on the membrane resistance (R) or conductivity (1/R). Note, however, that the value of R [111,146], which is found from the slope of the initial section of the CVC (Figure 6), includes not only the IEM resistance and the resistance of the foulant layer on its surface, but also the resistance of the solution between the Luggin capillaries. To exclude the effect of this solution on resistance, it is necessary to obtain a CVC under similar conditions, but without a membrane, or use other methods for measuring the electrical resistance of membranes, which are reviewed in [70,72]. 

The length of the inclined plateau in CVC (Region II in Figure 6b), or rather, the potential drop between points *a* and *b*, determined by the intersection of tangents to Region I–Region II and Region II–Region III in CVC, can correspond to the onset of nonequilibrium electroconvection, which develops according to the Rubinstein–Zaltzman mechanism [152,153]. The ratio of the conductivities (1/R) determined from the initial (Region I in Figure 6b) and over-limiting (Region III in Figure 6b) sections of the CVC is an indicator of the degree of EC and/or WS development, in particular, due to the appearance of a foulant layer on the membrane surface [111,146,154,155]; the conductivity of the membrane system increases in an over-limiting state (R_3_/R_1_ < 1) due to the development of WS and EC. Comparing the “plateau length” and R_3_/R_1_ values of pristine and fouled membranes, it is easy to assess the effect of foulants on WS and EC. 

Point *a* (Figure 6) corresponds to the experimental value of the limiting current density, *i_lim_*^*exp*^. According to the Peirce equation [117], modified by the Seung-Hyeon Moon group [156], this current is determined by the ratio of the limiting current, $I_{\lim c}^{exp},$ to the conducting (not screened by foulant) IEM surface, $S_{c}$:

(18) $$i_{lim}^{exp}=\frac{I_{\lim c}^{exp}}{S_{c}}=\frac{FD_{1}C_{1}}{\delta }\left(1-\frac{z_{1}}{z_{A}}\right)=\frac{FDC}{\delta \left(T_{1}-t_{1}\right)}$$

where $S_{c}=\varepsilon S$; *S* is the IEM surface polarized by electric current, ε is the surface fraction not occupied by the foulant (or other nonconducting substance); *D*_1_, *C*_1_, *t*_1_ are the diffusion coefficient, concentration and transport number of the counterion in the solution; *z*_1_ and *z*_A_ are the electric charges of the counterion and co-ion, respectively; *D* and *C* are the diffusion coefficient and the concentration of the electrolyte in the solution; *T*_1_ is the counterion transport number (determined by Equation (1); and *δ* is the depleted diffusion layer thickness.

Thus, from the value of *i_lim_*^exp^ found by graphical treatment of CVC (Figure 6b), it is easy to estimate the effect of foulant on the depleted diffusion layer thickness if the counterion transport numbers in the pristine and fouled membranes are known, or to solve the inverse problem [157]. In particular, in articles [111,146], *T*_1_ was found as the difference 1 – *T*_W_. The values of *T*_H_ (for CEM) and *T*_OH_ (for AEM) were determined by the Hittorff method, i.e., using the change in the concentration of water splitting products in the desalination compartment and adjoining concentration compartments of electrodialyzer. Equation (18) also makes it possible to find the real limiting current density on the conducting areas of the IEM surface, if the value of ε is known. Using the proposed algorithm, Park et al. [146] concluded that bovine serum albumin, BSA (whose molecules in the working solution were charged negatively), does not enter the electrostatic interactions with the negatively charged CMX surface, but, nevertheless, partially screens it. Since BSA contains aromatic amino acids [158,159], it can be assumed that the reason for this screening was π-π (stacking) interactions and the formation of hydrogen bonds between the membrane surface and the foulant [56,160]. Electrostatic interactions between the positively charged AMX surface and the negatively charged BSA species cause more significant screening of the AEM surface than the CEM surface [146]. The formation of a bipolar boundary (see insert on the Figure 6a) is facilitated by WS, whose products (H^+^ and OH^–^ ions) have high diffusion coefficients as compared to other components of the processed solution. These H^+^ and OH^–^ ions reduce the conductivity of fouled membrane and ajoining solution in comparison with that observed in the case of the pristine AMX membrane. In addition, Park et al. [151] found a 30% increase in the diluted diffusion layer thickness at the surface of BSA-fouled AMX membrane as compared to the pristine one, as well as fouled and pristine CMX membranes. Besides, *i_lim_*^*exp*^ increased after membrane fouling by BSA in the case of the CEM, but decreased in the case of the AEM (Figure 6). The authors of [151] did not explain these changes, because the understanding that foulants can significantly affect the surface properties responsible for the development of EC and WS, and that the equilibrium EC can affect the *i_lim_*^*exp*^ value [161,162], began to take shape only in recent years.

**Figure 7:**
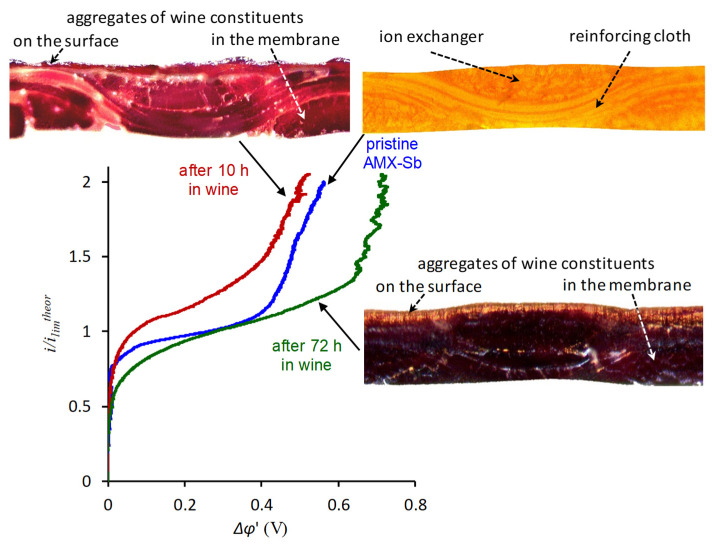
Corrected current–voltage characteristics after ohmic component subtraction, obtained in 0.02 NaCl solution for pristine and fouled in red wine AMX-Sb membrane samples. The insets show optical images of cross-sections of these samples. The value of *i_lim_*^theor^ was calculated using the Lévêque equation [163]. Adapted from [64].

To reveal this effect, along with CVC measurements, it is required to know the chemical composition of foulants, water contact angle and zeta potential, which allow estimation the degree of hydrophobicity and the magnitude of the surface charge. The use of 2D and 3D SEM, AFM, SECM and various types of optical microscopy provides insight into the electrical and geometric inhomogeneity of the IEM surface, as well as (analysis of IEM cross section) into the foulant film thickness. For example, the use of these methods along with the CVC measurements (Figure 7) and the simultaneous recording of the pH difference at the inlet and outlet of the desalination channel (which contained the pristine or fouled in red wine AMX-Sb membrane), made it possible to establish that the degree of WS and EC development is determined by the time of the AEM contact with the foulant [64]. 

Thus, after 10 h of contact with wine, the ohmic resistance of AMX-Sb doubles. At the same time, WS decreases, EC intensifies, and mass transfer by chloride ions increases in comparison with the pristine membrane. The reason is the formation of the island-like structure of the more hydrophilic foulant layer on the more hydrophobic pristine membrane surface. Moreover, foulant aggregates (colloidal structures of anthocyanins, tannins, polysaccharides and other wine components) have a lower conductivity than the pristine membrane. The formation of such an electrical inhomogeneity, which is accompanied by an increase in the geometric heterogeneity of the surface, is the reason for the appearance of EC, which develops according to the mechanism of electroosmosis of the first kind [161]. This type of EC contributes to an increase in the limiting current density, *i_lim_*^*exp*^, found using the CVC graphical treatment. Note that theoretical estimates made for the case of natural convection of the feed solution [139] predict the maximum development of EC if 50% of the membrane surface is screened by a non-conductive substance. For forced convection of the feed solution, both theory [141] and experiment [140] give the maximum development of EC if 10–20% of the IEM surface does not conduct an electric current.

An increase in the time of contact with wine leads to an enrichment of the volume and surface of AMX-Sb with polyphenols. The result of this enrichment is a sharp drop in the electrical resistance of the fouled samples in comparison with the pristine membrane and the formation on its surface of an almost continuous layer of foulant, 2–3 µm thick. Moreover, the electric charge of the foulant surface is much lower than that of the pristine membrane. The result of such surface changes is a weakening of EC and enhancement of WS.

Belashova et al. [164] applied the same complex approach in studying the consequences of precipitation after the operation of the CMX-Sb cation exchange membrane (Astom, Osaka, Japan) in a solution (pH = 12) containing Na_2_CO_3_ (1000 mg/L), KCl (800 mg/L), CaCl_2_ (800 mg/L) and MgCl_2_ (452 mg/L), and in the same solution from which Na_2_CO_3_ was excluded to prevent the formation of carbonate precipitates. The precipitate formation was carried out in an over-limiting current mode. Simultaneously with CVCs obtained in 0.02 M NaCl solution, pH differences were measured in the ED desalination channel formed by the pristine or fouled membrane under study. Precipitates on the membrane surface were studied by SEM, EDS and X-ray diffraction analysis (XRD) of the surface. These researchers showed that the water splitting intensity is influenced by the localization of substances in the precipitate layer. In particular, in the case of deposition of magnesium hydroxide and calcium mixture on the CMX-Sb surface, WS is enhanced in comparison with the pristine membrane. The appearance of a dense film of calcium carbonate on top of this mixture prevents the contact of hydroxide ions with water molecules. As a result, water splitting is suppressed and EC increases.

### 5.3. Chronopotentiometry

Chronopotentiometry is another rapidly developing method. It consists of applying a direct electric current to the membrane system and recording a potential drop in the same electrochemical cells as in the case of CVCs. A review of the possibilities of this method, including its application to assessment the effect of inorganic and organic foulants on membrane behavior in an electric field, is described in detail in a recent review [83].

This method is most widely used [146,165,166,167,168,169] to determine the fraction of the conducting surface *ε*, which the Seung-Hyeon Moon group proposed to find [155,156] by the value of the transition time, *τ*, in the initial section of chronopotentiogramm (ChP), using the Sand equation adapted for IEM [170]:

(19) $$\varepsilon =\frac{2i\tau^{1/2}\left(T_{1}-t_{1}\right)}{Cz_{1}F\left(\pi D^{1/2}\right)}$$

However, in later works, Mareev et al. [171] showed that Sand equation (which was used to determine *ε*) is applicable to real IEMs only when the limiting current is exceeded by a factor of 1.5–2. This limitation is due to the fact that the Sand equation assumes the direction of streamlines strictly perpendicular to the membrane surface. This condition is met only if the dimensions of the electrical inhomogeneity of the surface are commensurate with or exceed the depleted diffusion layer thickness. This is why SEM, SECM and optical microscopy appear to be more reliable methods for determining *ε*.

**Figure 8:**
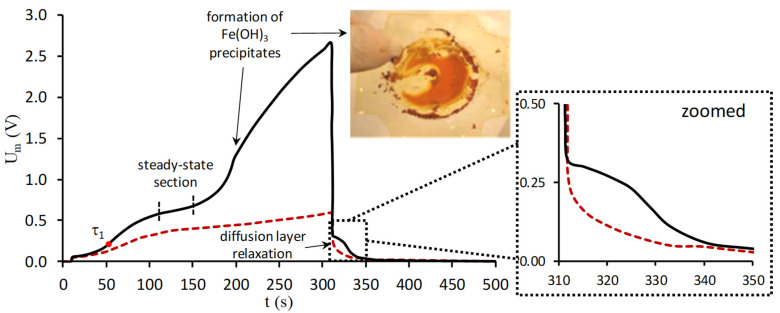
Chronopotentiometric curve of Nafion-117 membrane in 0.01 M Fe_2_ (SO_4_)_3_ solution in overlimiting current regime. Dashed line shows a typical shape of the curve in NaCl solution. The inset shows the precipitate found on the studied membrane surface after obtaining the chronopotentiometric curve (optical image). Adapted from [176].

Note that the possibilities of chronopotentiometry are not limited to the conducting surface fraction assessment. The ChP analysis makes it possible to follow up (including on-line) the effect of foulants on the counterion [83] and co-ions transport numbers in the membrane phase [172]. In addition, chronopotentiometry allows determination of the current densities at which intensive fouling or scaling of membranes begins, and estimation of the time interval required for the start of this process [123,173,174,175]. In the case of precipitation or foulant layer formation on the IEM surface, the ChP takes a special shape. In particular, an increase in the potential drop takes place some time after reaching the steady-state [123,174,175] (Figure 8). This increase is caused by an amplification in the concentration polarization of the membrane system due to a decrease in the IEM conductive surface fraction caused by precipitation. A decrease in the potential drop after switching off the current and an increase in the time required for the diffusion layer relaxation as compared to the pristine membrane (Figure 8) are other indicators of the precipitate presence on the surface [123,173,174,175]. Apparently, this change in the ChP shape is due to chemical reactions between substances that are part of the precipitates (for example, hydroxides of magnesium, iron and other metals) and WS products (for example, protons in the case of a CEM), which are released from the IEM at the moment the current is switched off.

In cases where the influence of the surface electrical heterogeneity on the transition time value can be neglected, the *i*τ^0.5^ − *i* (Sand coordinates) dependence is a straight line parallel to the abscissa axis if the counterion transport in the membrane system is carried out under diffusion control. A decrease in the experimental values of *i*τ^0.5^ in comparison with the values calculated by the Sand equation sometimes is an indicator of the conversion of the counterions of weak electrolytes in the near-membrane solution into a molecular form. For example, this phenomenon is observed in the presence of an amino acid (lysine) [177] or phosphoric acid anions [178] in a solution due to the participation of these substances in protonation–deprotonation reactions. The sloped line of the same dependence indicates the limitation of mass transfer by chemical reactions that occur in the near-membrane solution or on the IEM [179] surface. The appearance of an additional inclined section with another transition time on the ChP is characteristic of AEMs that operate in solutions containing phosphoric acid anions, polybasic carboxylic acids [132,154,178] or ammonium cations [77,180]. The presence of such a section is an indicator of a shift in the AEM internal solution pH to the alkaline values. Such a shift results in the formation of concentration profiles in the membrane of multiply charged ions of inorganic or organic acids [132,154,178], as well as NH_3_ molecules [77,180]. This knowledge allows us to predict the prospects for IEM fouling by similar substances, which are often found in liquid media of the food industry.

### 5.4. Electrochemical Impedance Spectroscopy

Electrochemical impedance spectroscopy (EIS) is a very useful method for studying transport phenomena in membrane systems [132,181,182,183] and various aspects of IEM fouling by components of food production liquid media [21,144]. Measurements of electrochemical impedance spectra can be carried out in the same electrochemical cells as in the case of CVC and ChP. Recording of impedance spectra is carried out at a given direct current, as well as an alternating current (AC) having a significantly lower amplitude. As a rule, the overall AC frequency range is from 10^−3^ Hz to 10^5^ Hz.

Park et al. [146], were apparently the first to use EIS to identify the WS intensity in the pristine and BSA-fouled IEM by analyzing the parameters of Gerischer impedance arc.

**Figure 9:**
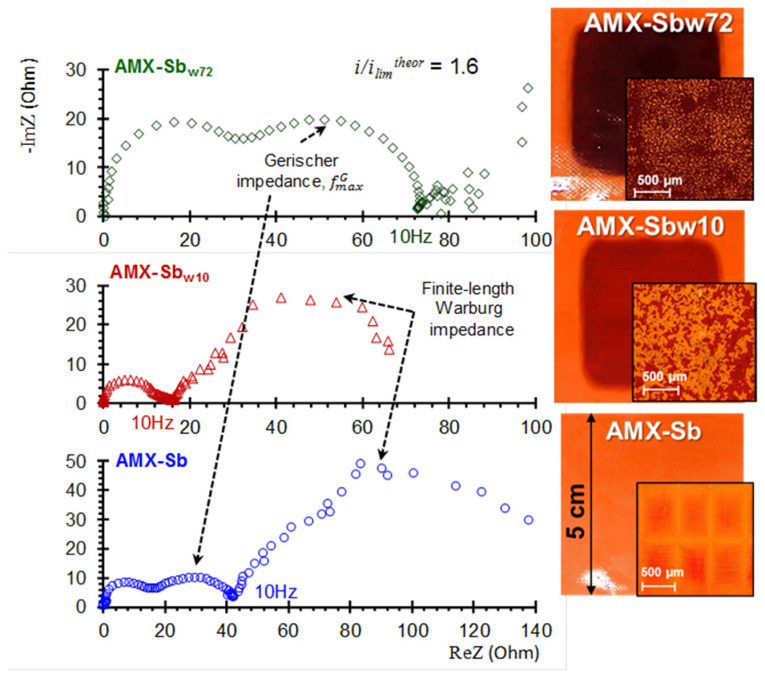
Electrochemical impedance spectra of pristine anion exchange membrane AMX-Sb and the same membrane after contact with wine for 10 (AMX-Sb_w10_) and 72 (AMX-Sb_w72_) hours. The data were obtained in 0.02 M NaCl solution; on the right are optical images of the studied samples. Adapted from [62].

In work [62], a brief overview of modern studies devoted to the use of EIS is given and, using the example of AMX-Sb samples in contact with wine, the capabilities of this method are demonstrated. In particular, it was shown that the foulant island-type layer (formed after 10 h of AMX-Sb contact with wine; sample AMX-Sb_w10_, Figure 9) contribution to an increase in the fouled membrane electrical resistance does not exceed 15%. Moreover, the presence of this membrane in a 0.02 M NaCl solution during the impedance spectra measurement decreases this contribution, apparently, due to the partial destruction of colloidal structures in the foulant layer.

The value of the foulant layer electric capacitance increases by two orders of magnitude as compared to the pristine membrane. These data were obtained by analyzing the high-frequency impedance spectra (or, rather, the superposition of the locus that characterizes the membrane and the arcs that characterize the foulant layer) using the electrical equivalent circuits method. Such an analysis is used, for example, for bilayer IEMs or membranes with modified surfaces [184,185,186], as well as in situ monitoring of membrane fouling in the course of natural water ED [80,81].

The treatment of low-frequency impedance spectra (Warburg-type impedance) using the previously developed concepts [187,188] made it possible to calculate the depleted diffusion layer thicknesses, and to establish that in overlimiting current modes this thickness decreases with an increasing current. Moreover, this decrease is most significant in the case of the AMX-Sb_w10_ as compared to the pristine AMX-Sb membrane. These results confirmed the development of more intensive equilibrium and non-equilibrium EC in the case of AMX-Sb_w10_, of which the surface contains island-type distributed foulants.

In the analysis of the medium-frequency impedance spectra (Gerischer-type impedance) of the pristine and wine-contacted membranes, model concepts were applied, which were developed to determine the effective WS constant in systems with bipolar and monopolar membranes [189,190]:

(20) $$\chi =\frac{2\pi f_{max}^{G}}{\sqrt[{}]{3}}$$

Equation (20) allows estimation of the effective constant of the WS reaction *χ* if the value of $f_{max}^{G}$ (frequency corresponding to the maximum in the Gerischer impedance spectrum) is known. These estimates showed that the sizes of the Gerischer arc (Figure 9) and the *χ* values increase in the series: AMX-Sb_w10_ < AMX-Sb << AMX-Sb_w72_.

The combination of EIS with biochemical analysis of the studied samples showed [62] that the intensification of WS in the case of the AMX-Sb_w72_ is facilitated by biofouling. Water splitting intensification is explained by the presence of native structures (membranes, RNA, etc.) in microorganisms, which contain, for example, phosphonate groups, characterized by very high catalytic activity with respect to WS reaction [10,126].

**Figure 10:**
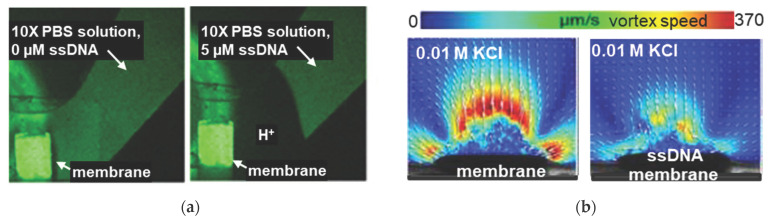
Results of visualization of the front of protons formed due to water splitting (**a**) and electroconvective vortices (**b**) at the surface of the anion-exchange membrane. On the left is the pristine membrane; on the right is a membrane on the surface of which single-stranded DNA (ssDNA, which contains dissociated phosphonate groups) is adsorbed. Adapted from [191,192].

Note that in recent years, electrochemical methods traditionally used to study the fouling effect on the behavior of membrane systems in an electric field have been supplemented with modern tools for WS and EC visualization. For example, Slouka et al. [191,192], using optic microscopy with fluorescein and rhodamine dyes (Figure 10), proved the ability of RNA adsorbed on the IEM surface to intensify WS and suppress EC.

**Figure 11:**
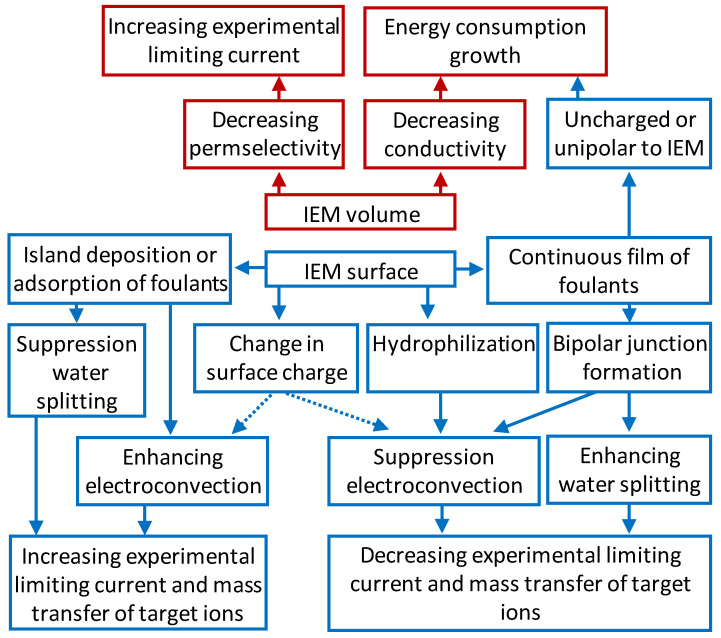
The scheme of the effect of fouling of the volume and surface of ion-exchange membranes on their behavior in an electric field.

Figure 11 summarizes the effect of volume and surface fouling of ion exchange membranes on their behavior in an electric field.

## 6. Non-Destructive Methods of Membrane Fouling Control

The detrimental effect of traditional cleaning agents (acids, alkalis and oxidants) on the equilibrium and transport characteristics of membranes (see Section 2, Section 3 and Section 4) contributes to the search for alternative, non-destructive methods of fouling remediation. The application of these methods is based on knowledge gained from studying the mechanisms of IEM fouling in an electric field and in its absence. Note that the frequency of cleaning must also be carefully considered to avoid disrupting the separation process. There are so many different problems, each of which requires an individual answer depending on the materials of which the membranes are made, the type and composition of the solution being treated, as well as the hydrodynamic and electrical modes of electrodialysis. These problems, traditional and alternative cleaning procedures and the peculiarities of its implementation, depending on the chemical nature of the membranes, as well as the component composition of the processed liquid media, were discussed in detail in a recent review [193]. Therefore, below we will only give a few examples of this diversity. 

The exposure to solutions of NaCl or other salts leads to partial destruction of colloidal particles on the surface and inside the IEM due to the salting-out effect. The higher the salt concentration in the regenerating solution, the closer the regenerated membrane conductivity to the conductivity of the pristine membrane [60,61,105]. The removal of colloidal particles destruction products from the IEM pores is apparently limited by their diffusion towards the external solution; the thinner the membrane and the larger its pores, the faster and more completely the regeneration process proceeds. Nevakshenova et al. showed [105] that cleaning of an AEM after its contact with wine using concentrated (58.5 g/L, pH 5.5) NaCl solution allowed almost completely restoration of AEM selectivity if the duration of its contact with wine does not exceed 70 h. In the case of a thin (140 ± 10 μm) homogeneous AMX-Sb membrane, the transport numbers of the Cl^–^ counterion in the samples of pristine and regenerated membranes differ by less than 1%. In the case of a thick (530 ± 10 µm) heterogeneous membrane MA-41P (OJSC Shchekinoazot, Pervomaisky, Russia), this difference increases to 3%. At the same time, the use of saline solutions for IEM regeneration, which have worked for a long time in industrial wine stabilization processes and have already been cleaned with acids and/or alkalis, gives less encouraging results [60,61]. For example, the conductivity of such CEMs and AEMs (Astom, Osaka, Japan) reaches, respectively, 60% and 45% of the pristine membranes conductivity, and the value of $f_{2}$ can be increased, respectively, by 12% and 23%. The fact is that NaCl solutions with neutral pH values have little effect on the electrostatic and π-π (stacking) interactions of polyphenols with ion-exchange membranes. 

These solutions are not able to destroy agglomerates of polyphenol-containing nanoparticles that are formed in the pores of IEMs due to cleaning with acids and alkalis. The use of regenerating solutions that contain more hydrated Ca^2+^, Mg^2+^, and SO_4_^2−^ ions could provide a more complete destruction of colloidal particles, as is observed, for example, in the case of regeneration of ultrafiltration membranes, used, for example, in BSA separation [194]. Meanwhile, after the use of such solutions, the conductivity of CEMs and AEMs does not increase, but decreases in comparison with conductivity achieved after prolonged ED stabilization of wine [60,61]. The reason for this decrease is the specific interactions of Ca^2+^ ions (and to a lesser extent Mg^2+^) with fixed sulfonate groups of cation-exchange membranes, which lead to a loss in their exchange capacity and a decrease in the surface charge [172], as well as the formation of complexes with polyphenols by these ions [195]. 

Application of acidified (pH = 3.5) aqueous ethanol solution (12%) recovers IEC by 33% and nearly doubles the conductivity of CEMs and AEMs fouled with polyphenol. This result is apparently due to the destruction of hydrogen bonds in the presence of ethanol. Moreover, a more noticeable regeneration is achieved in the case of AEMs due to the acquisition of a positive electric charge by polyphenols in an acidic medium, as well as due to the electrostatic repulsion of these particles from the positively charged fixed groups of the anion-exchange membrane. 

The use of a regenerating solution that contains equal volumes of acetonitril, methanol, isopropyl and distilled water allows an even more complete recovery of phenolic monomeric and polymeric substances (more than 30 items) from CEMs and AEMs that were in contact with wine [26] or cranberry juice [23]. Such recovery is due to the destruction of hydrogen bonds and π-π (stacking) interactions of polyphenols with the material of IEMs, as well as with proteins, amino acids, saccharides and other substances found in juices and wine. As expected, a more complete recovery of phenolic substances is achieved in the case of membranes having an aliphatic matrix (CEM Type-ll, CJMC-5) [23]. 

Regeneration of both the aromatic (CSE-fg, MK-40) and aliphatic (CEM Type-ll, CJMC-5) cation exchange membranes is facilitated by alkalization of an organic solvents mixture to pH = 10. The result of such alkalization is the initiation of electrostatic repulsion of negatively charged sulfonate fixed groups of IEMs and polyphenols, which acquire a negative charge. However, the use of this solution, as well as of saline solutions, does not provide complete regeneration of ion-exchange membranes. Therefore, the question about the optimal solution for the mild chemical regeneration of IEMs after their contact with juices, wines, tea and other phenolic-containing liquid media of the food industry remains open.

Another promising method for the regeneration of IEMs after their contact with liquid media of the food industry is the use of enzymatic agents specific to certain types of substances. They performed well in the case of cleaning of ultrafiltration membranes fouled by proteins [196] or in the case of reducing membrane fouling in bioreactors [197]. Bdiri et al. [198] first tested protease and β-glucanase, as well as polyphenol oxidase, which enter the specific interactions with proteins, polysaccharides, and phenolic compounds, respectively, as well as mixtures of these enzymes. They showed that the use of these cleaning agents can increase the conductivity and exchange capacity of the wine-fouled CMX-Sb cation-exchange membrane by at least 25% and 12%, respectively. The most encouraging results have been obtained with Tyrosinase, which is active against polyphenols.

All previous examples dealt with the remediation of fouling phenomena. However, another alternative approach exists, and consists of preventing fouling using surface modifications or special hydrodynamic and electrical modes of electrodialysis [3,199]. The most studied is the use of pulsed electric fields (PEF) to prevent fouling in the processing of whey and similar liquids [3,200]. In some works [201], there is evidence of a decrease in energy consumption and a decrease in fouling during ED separation of citric and malic acids from cranberry juice. Fouling by organic and inorganic substances when using PEF can be prevented due to a decrease in concentration polarization in the membrane system, a decrease in pH changes in near-membrane solutions, and ensuring favorable conditions for the development of electroconvection. The main advances in this field are comprehensively reviewed in a recent work by Bazinet and Geoffroy [3]. Therefore, we will not focus on this topic.

As to surface modification, it may be chemical or geometrical. Many kinds of chemical modification have been proposed in order to improve IEMs anti-organic fouling properties in recent studies (see for example [202,203,204]). Most of them focus on increasing the hydrophilicity of the AEMs surface [202] and imparting an electrical charge, which is the opposite to that of the AEMs fixed groups [203,204]. Extensive reviews on the modification of commercial membranes, as well as synthesis of selective and anti-organic fouling IEMs, can be found in [205,206,207,208].

The geometrical method consists of creating profiles of a few hundred micrometers on the surfaces of the IEMs in order to increase the exchange area, to enhance the hydrodynamic turbulence phenomena and to stimulate electroconvection [209]. These modification reduce concentration polarization and/or enhances mixing of the solution at the IEMs surface, preventing the formation of fouling layers. Details of membrane surface profiling can be found in the review [210].

## 7. Conclusions

The study of the consequences of ion-exchange membrane fouling and the development of methods of fouling control in dialysis and electrodialysis processes of food industry liquid media processing is increasingly attracting the attention of scientists.

In recent years, a certain algorithm for studying the characteristics of ion-exchange membranes has been developed, which includes comprehensive studies of exchange capacity, water content, conductivity, diffusion permeability before and after the operation of membranes in industrial or laboratory dialysis and electrodialysis processes. To interpret these data, the microheterogeneous model is increasingly being used. The recent modifications of this model take into account the possibility of formation in the pores of IEMs of hydraulically permeable (dairy industry) or impermeable (wine and juice industry) agglomerates of colloidal particles, the surface charge of which is identical or opposite to the electric charge of the pore walls. The use of this and a number of other known models makes it possible to estimate changes in the structural parameters of IEMs, the diffusion coefficients of counterions and coions, as well as the transport numbers of these ions (membrane selectivity) caused by interactions of foulants with the polymer matrix and fixed groups of IEMs.

The behavior of fouled IEM in an electric field (i.e., in electrodialysis processes) is largely determined by changes in electrical resistance, hydrophilic/hydrophobic balance, as well as the roughness of its surface and the foulants distribution morphology on this surface. These characteristics drive the development of electroconvection (which promotes stirring of the solution and counteracts fouling) and water splitting (which often enhances fouling due to local pH changes at the surface of the IEMs and within the membrane).

To study these phenomena, voltammetry, chronopotentiometry and impedance spectroscopy are most widely used. Moreover, the electrochemical impedance spectra obtained in the presence of a direct current in the membrane system provide the most complete information, including the resistance and thickness of foulant layers on the membrane surface, effective reaction rate constants of H^+^ and OH^–^ ions generation, as well as the thickness of diffusion layers, which depends on the degree of electroconvection development. The use of modern models describing the behavior of membrane systems in overlimiting current modes has shed light on the reasons for the enhancement of water splitting during IEM fouling by proteins, polyphenols, or biofouling. It turned out that in some cases (island localization of polyphenols on the IEM surface), fouling can promote the increase in useful mass transfer due to the intensification of electroconvection.

The idea was finally formed that traditional IEM cleaning methods using acids, alkalis and/or oxidants are not only harmful to the environment, but also contribute to the destruction of membranes and an increase in fouling. Mild chemical reagents (aqueous salt solutions, ensims, mixtures of polar and non-polar organic solvents and water), as well as controlling the membrane regeneration process by varying the solution pH, seem to be more promising along with the use of a specially designed anti-organic fouling membranes.

In order for the results of an experimental study of the foulants effect on the structure, transport and mass transfer properties of IEMs to be more informative, it is necessary to improve the existing and develop new mathematical models that will more deeply take into account the nature of the foulants interactions with materials from which membranes are made, as well as consider the specifics of many components of food industry liquid media; the ability to change the structure and electrical charge depending on the environment pH.

Another challenge is to develop environmentally sound ways to counter biofouling and find solutions for the regeneration of fouled membranes (for example, using enzymes specific for key toxic substances), as well as to determine the optimal current regimes that will ensure suppression of fouling, low energy consumption and high current efficiency in ED processes in the food industry. The use of pulsed electric fields seems to be the most promising for achieving this goal.

## Figures and Tables

**Table 1:** Parameters of the modified microheterogeneous model [60] calculated for the AEM samples soaked with the synthetic polyphenol containing solution.

AEM Soaking Duration, h	*f*_2app_	*f*_2_	*f*_2s_	*f*_cp_	$D_{-}^{g}$ $/D_{-}^{g}\;\left(h\;=\;0\right)\;$	d/d (h = 0)	IEC_sw_ (mmol cm^−3^)
0	0.11	0.09	0.09	0	1.00	1.00	2.30
24	0.087	0.09	0.07	0.02	1.26	1.02	1.95
100	0.084	0.12	0.07	0.05	1.44	1.05	1.84
500	0.073	0.16	0.06	0.1	1.47	1.08	1.77
750	0.071	0.18	0.05	0.13	1.74	1.08	1.72
1000	0.069	0.20	0.045	0.155	1.88	1.08	1.70

## Data Availability

Not applicable.

## Abbreviations

All other abbreviations have their usual meaning, or are sufficiently explained in the text.

AEM	Anion-exchange membrane
BSA	Bovine serum albumin
CEM	Cation-exchange membrane
ChP	Chronopotentiogramm
CVC	Current–voltage characteristic
EC	Electroconvection
ED	Electrodialysis
EDS	Energy dispersive X-ray spectrometry
EIS	Electrochemical impedance spectroscopy (spectrum)
FO	Forward osmosis
IEC	Ion exchange capacity
NF	Nanofiltration
PEF	Pulsed electric field
PP	Polyphenol
PVC	Polyvinyl chloride
RNA	Ribonucleic acid
RO	Reverse osmosis
SECM	Scanning electrochemical microscopy
SEM	Scanning electron microscopy
UF	Ultrafiltration
WS	Water splitting
XRD	X-ray diffraction
